# Association of *BRCA1 185 del AG* with early age onset of breast cancer patients in selected cohort from Pakistani population

**DOI:** 10.12669/pjms.345.15764

**Published:** 2018

**Authors:** Durr-e-Samin, Muhammad Saif-ur-Rehman, Muhammad Shahnawaz-ul-Rehman, Muhammad Sajjad Khan

**Affiliations:** 1 Durr-e-Samin, Ph.D Candidate. Centre of Agriculture Biochemistry and Biotechnology, University of Agriculture, Faisalabad, Pakistan; 2 Muhammad Saif-ur-Rehman, Ph.D. Institute of Animal and Dairy Sciences, University of Agriculture, Faisalabad, Pakistan; 3 Muhammad Shahnawaz-ul-Rehman, Ph.D. Centre of Agriculture Biochemistry and Biotechnology, University of Agriculture, Faisalabad, Pakistan; 4 Muhammad Sajjad Khan, Ph.D. Institute of Animal and Dairy Sciences, University of Agriculture, Faisalabad, Pakistan

**Keywords:** Breast Cancer, Pakistani population, *BRCA1*, *Exon-2*, *185-Del-AG*, *185 Ins. A*

## Abstract

**Background & Objective:**

Large spectrum of pathogenic *BRCA* mutations is known as a major cause of hereditary breast ovarian cancer in human all over the world. The objective of present study was to find out the association of mutations*185-del-AG* and *185Ins.A* at *BRCA1* exon-2 with age of onset and family history of gynecological cancer among the selected cohort of breast cancer patients in Pakistani population and to provide guidelines for treatment strategies.

**Methods:**

For the present study 115 subjects were recruited from different hospitals of Punjab, Pakistan, during May, 2017 to February, 2018. The inclusion criteria were age ≥30, without any previous *BRCA* testing and willingness to participate in present study. Subjects were interviewed for various demographic factors. Out of 115 subjects, 46 were selected on the basis of findings of previous studies and approximately 3 ml of blood was collected in EDTA coated vials for analysis of BRCA1 exon-2. Column based DNA extraction was performed by using commercial kit and exon specific primers were used to amplify *BRCA1* exon 2 and PCR products were sent for sequencing to Eurofins Genomics. Sequences were analyzed through the BLAST program at National Center for Biotechnology Information (NCBI) and Bio Edit software. Accession numbers were obtained on submission of sequences in GenBank.

**Results:**

*BRCA1-185-del AG mutation was found in one of the breast cancer patient who was 33 years of age at diagnosis*. None of the samples revealed positive results for *BRCA1-185 Ins. A*.

**Conclusion:**

*BRCA1-185 Del AG* mutation has association with early age onset of breast cancer. The direct sequencing is very useful approach for *BRCA* analysis and exon specific selected cohort from Pakistani population.

## INTRODUCTION

The DNA repair associated breast cancer gene1 (*BRCA1*, BIC - Gene ID: 672, position: 43044295 to 43125483bp) in human being, is located on ‘q’ arm of chromosome 17(17q21.3), spread over 81 kb of genome, with 24 exonic regions and produces 7.8kb of mRNA which translate into type 1 breast cancer susceptibility protein.^1^ *BRCA1* translation products contain numerous important motifs which have strong interactions with other proteins involved in the processes; like progression of cell cycle, responses to DNA damages, maintenance of genomic integrity, ubiquitination and apoptosis.^2^ *BRCA1* gene products are very important transcriptional regulators having vital role in repair of double strand DNA breaks mediated by homologous recombination.^3^ Germline mutations, in *BRCA1* are also associated with male breast, uterine, cervical, pancreas and prostate cancers beside breast/ovarian carcinomas.^4^ It has been estimated that in human female, cumulative frequency for mutations in this gene imposes a risk of 87% of breast and 50% of ovarian cancer.^5^,^6^ The *BRCA* databases reported over 6000 variants among which around 1800 have been classified as likely pathogenic or pathogenic in nature.^7^ The frame shift mutations named;*185-del AG (c.68_69delAG-Allele ID 32701) and185 ins.A (c.66 dup A- Allele ID: 46247)*are well established pathogenic variant at exon 2 of *BRCA1* gene. The first one occurs by deletion of AG as a consequence of which, stop codon appears after few base pairs that results in significant truncation of protein.^8^ The *185-del AG* mutation was first time described as founder mutation in Ashkenazi Jews population.^9^

The rapid rise in the incidence of carcinomas of gynecological origin in Pakistan is imposing challenge to expand investigations on *BRCA* mutations to provide guidelines to determine treatment strategies. Since according to previous studies, one of the commonly found *BRCA1* aberrations in Pakistani population is *185delAG* 1.^10^ Thus, the present study was aimed to investigate incidence of this mutation along with another well-established *BRCA1*variant at exon 2 and relationship of these with age of onset and family history in Pakistani population. The latest development in research explored the importance of *BRCA1/2* mutations for determining treatment regimes. In the recent years, Food and Drugs Administration has approved PARP (Poly adenine dinucleotide phosphate polymerase) inhibitors such as olaparib to treat certain tumors having *BRCA1* mutation.^11^,^12^ Due to administrations of such recommendations in clinical practice the trend of *BRCA* testing is increasing.

## METHODS

Present study was carried out at Centre of Agriculture Biochemistry and Biotechnology in the University of Agriculture, Faisalabad, Pakistan. Research was approved by institutional Biosafety/Bioethical committee through the Office of Research, Innovation and commercialization (IBC, ORIC, UAF, Pakistan). A total of 115 subjects (85 cancer patients, 30 non-cancerous) were selected from different hospitals of Punjab, Pakistan. The subjects were interviewed for personal health and reproductive features such as age at menarche, menopause and first child birth, total number of children parental consanguinity and ethnicity. Beside that family history of cancer of gynecological origin or any other kind of cancer was also recorded. Regarding this, positive family history was defined for present study as having at least one first degree or two second degree relatives with gynecological cancer, and negative family history as none of first or second degree relative with such cancer (excluding themselves in case of patients). Demographic features were recorded on SPSS version 4. Age patterns and some other features were compared with previous studies done on Pakistani population.

### Inclusion criteria

The inclusion criteria were ≥30 age at collection of data/blood sample (except for a few cancer patients who presented with the disease before this age), no *BRCA* test before this study and willingness to participate in research by informed consent. Subjects were never forced to provide any kind of information.

### Selection of samples

Blood samples of 46 out of 115 female subjects selected on the basis of age at sampling/onset of disease and family history, for sequence analysis of *BRCA1*exon-2.

Subjects were classified in the following four groups.

1. Breast cancer patients with (N= 16)
  < 40 years of age at diagnosis
2. Breast cancer patients with (N=16)
  ≥40 < 60 years of age at diagnosis
3. Non- Cancer subjects with ≥30 and (N=7)
  <40 years of age
4. Non-Cancer subject with ≥40 (N=7)
  < 60 years of age

Regarding family history 80% (11/14) of non-cancerous subjects and 50% (16/32) of cancer patients were selected with positive family history according to definition for this study design. Selection criteria was formulated by careful examination of data from previous studies about analysis of *BRCA1* mutations at exon-2. The database for these two mutations with record of related explored features has been given in Appendix-I.

**Appendix-I:** Data set of mutations at exon-2 in Pakistani Population.

	Liede et al.(2002)^16^	Rashid et al. (2006)^17^	Malik et al. (2008)^19^	Moatter et al. (2011)^20^	Aziz et al. (2016)^18^	Present Study
No. of Patients^1^ (Ethnicity) Age at diagnosis(years)	1(OC) (Punjabi) 40	3(OC) (Punjabi) 41,<50, 57	0 - -	0 - -	0 - -	0 - -
No. of Patients^2^ (Ethnicity) Age at diagnosis(years)	1(BC) (Punjabi) 47	2(BC) (Pathan) 39, 40	0 - -	0 - -	1 (Punjabi) 35	1 (Punjabi) 33
Methods	Direct DNA sequencing PTT(For ex-11)	SSCP, PTT, DHPLC and DNA sequencing	SSCP	SSCP assay and DNA sequencing	Allele specific PCR	Direct sequencing of PCR products.
Exons	2,11,12,15,20	2,7,8,10,11,15,17,20,24	2,3,13	2,5,6,16,20,22	2,15	2
No. of Subjects	341(BC) 120(OC) 200(Control)	176	120	53	120	46
Family history of patient/proband with mutation	Negative family history for both mutations	Positive family history for all five mutations	Negative	-	Positive	Negative
Type of cohort studied	Case/Control	Familial	Unilateral, sporadic breast cancer patients with negative family history	Patients with moderate family history	Population based case control	Case/control with exon specific selection of cohort

1 Mutation 1: *BRCA1- 185 Ins. A*;

2 Mutations 2: *BRCA1-185 del AG,* BC: Breast cancer; OC: Ovarian cancer.

### DNA extraction and amplification

The blood samples (3 ml) were collected in EDTA vials with the help of medical professionals with care to avoid any discomfort or contamination. Extraction of DNA was done by using thermo scientific kit (K0781) by standard procedure according to instructions given by manufacturer. Following sequences of primer were selected as used by Singh et al. 2015.^13^

Forward primer
5’GAAGTTGTCATTTTATAAACCTTT3’
Reverse primer
5’GTCTTTTCTTCCCTAGTATGT3’

For PCR standard reaction mixture was prepared in 25ul volume for amplification of required DNA fragments, at primer annealing temperature of 51°C. The PCR products along with 250 base pairs ladder were run on 1.5% agarose in 0.5 X TBE to reveal amplified DNA fragment.

### DNA Sequencing

PCR products were sent for sequencing to Eurofins Genomics. FASTA files were analyzed by bioinformatics tools such as BLASTn, BLASTp. Analysis of chromatograms was done by using Bio Edit software. The wild type and mutant sequences were compared with *Homo sapiens* Taxon 9606, reference sequence at NCBI data base and submitted to GenBank by using BankIt browsing.

## RESULTS

### Demographic data analysis

Age at sampling/onset of cancer and family history were taken as criteria for analysis of *BRCA1*- exon 2 mutations in Pakistan ethnicity. The mean age at diagnosis of Breast cancer was 45±9.86 years (Ranged 26 to 65 years) with class boundaries shown in Table-I. Maximum number of Patients (63/85 -74%) fall in age ≥ 50 years. Regarding family history 73.91% (85/115) subjects provided required information among which 32.94% (28/85) from the patients while 53.33% (16/30) from non-cancerous subjects reported that they have positive family history according to definition of this study plan. None of other recorded feature produced any significant results.

**Table-I:** Age at diagnosis of Breast Cancer patient.

Class Boundaries (Years of age)	Frequency	Percentage
<40	27	31.77%
40 to 50	36	42.35%
51to 60	16	18.82%
>60	6	7.05%

Total	85	100%

### DNA extraction and BRCA1-exon-2 polymerization

**Fig.1:**
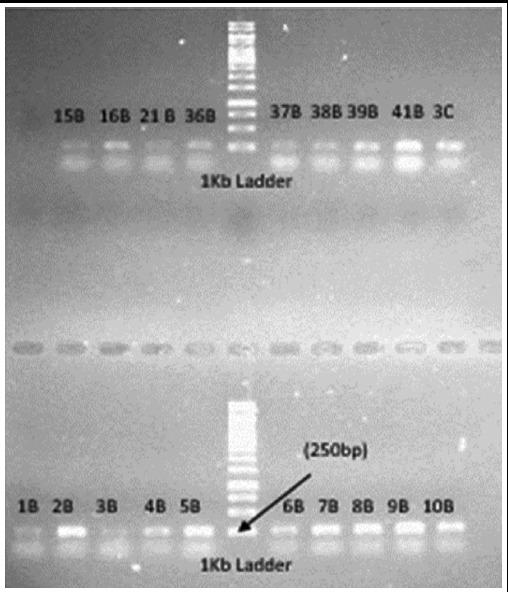
Amplified fragments of 258 base pairs.

DNA was successfully extracted, visualized on 1.5%agarose gel. PCR products resulted in synthesis of fragment of 258 nucleotide bases of Human *BRCA1*- exon 2 with flanking intron on both 5’ and 3’sides (Fig.1).

### Sequencing of DNA samples

PCR products sent for sequencing returned in FASTA files formats and chromatograms of the *BRCA1* exon 2 coding sequence of 99 base pairs, along with intronic flanking regions on both 3’ and 5’ ends. Sequenced fragments produced significant alignments on BLASTn and BLASTp and were verified from GenBank by obtaining following accession numbers: MH046834, MH046835, MH046836, and MH046837.

**Fig.2:**
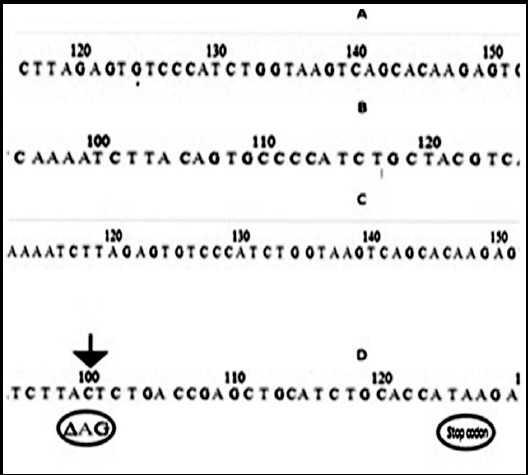
A, B, C: Wild type sequences of BRCA1-Exon-2. D: BRCA1-185 Del AG frame shift mutation with formation of stop codon.

*BRCA1-185-del AG* (*c.68_69delAG*) was found in the group-I (patients with age <40 years) in the breast cancer patient. Deletion and subsequent formation of stop codon (TAA) has been indicated at D, while A, B, C representing wild type sequences in Fig.2. The victim with mutation was diagnosed at the age of 33 years, having Punjabi ethnicity and reported negative family history for any kind of cancer. None of the sample was found to be positive for *BRCA1- 185 Ins. A (c.66dupA*).

## DISCUSSION

The well-known *BRCA1 -185 Del AG* pathogenic variant is one of the founder effect mutations whose incidence is variable among populations of different geographical and ethnic backgrounds.^14^ This variant also recognized in Pakistani population as one of the most common *BRCA1* alterations identified so far.^10^ In the present study design, the concept of deliberate selection of cohort for exon specific *BRCA* variants analysis for Pakistani population has been introduced so that most suspected individuals for different mutations could be screened. By using this approach, *BRCA1-185 Del AG* mutation was detected in 1/46 including all groups and 1/16 of most suspected group-I. None of the Sample was found to be positive for *185 Ins. A*.

The mean age of total of 115 subjects enrolled for present study was 46.55±11.01years while for the breast cancer patients mean age at diagnosis was 45±9.86 years. According to previous findings the 45 to 49 years range of age has significance in prognosis of breast cancer.^15^ To analyze variations at *BRCA1* exon 2, out of 115 subjects 46 were selected by narrowing the range up to five year from upper extreme (mean age 39.95±8.8). The victim of *185 del AG* was aged 33 at diagnosis this observation is in concordance with previous finding on this mutation among patients of cancer of gynecological origin with Pakistan ethnicity (Appendix-I). Regarding family history, patient in present study reported to have negative family history for any kind of cancer as Liede *et al.*, 2002^16^ also reported the victims with this mutation had negative family history for such cancers. Previous studies on Pakistani population also tried to find relation with ethnicity as Rashid et al., 2006^17^ reported BRCA1*- 185 Del AG in* two of the pathan patients while Liede *et al.*, 2006^16^ and Aziz *et al.*, 2016^18^ reported this mutation in patient with Punjabi ethnicity and currently it was also observed in patient with Punjabi ethnicity. In the two prior studies on Pakistani population that included exon-2 in investigation none of the patients found to be positive for *185 delAG*.^19^,^20^ The *BRCA1-185 Ins-A* mutation was also found in two of the previous studies that explored mutation in four ovarian cancer patients with Punjabi ethnicity.^16^,^17^ None of our selected samples revealed positive result for this mutation. The present study design has drawback of not including ovarian cancer patients for analysis of *BRCA1* at exon 2.

During the earlier studies, it has been revealed that a lot of heterogeneity existed in survival data and responses to cancer therapies between non-carriers and carriers and among carriers of different pathogenic variants of *BRCA1*. Recently during an experimental trial it has been found that *185 del AG* mutation exhibited sensitivity to platinum drugs and PARP (Poly ADP polymerase) inhibitors because of loss of ring domain in *BRCA1* protein.^21^ This feature of treatment sensitivity to double stranded breaks (DSB) inducing agents may serve as marker to prevent ineffective therapies and to develop some promising and unconventional therapeutic methods. Thus from current study it is concluded that *BRCA1 185 Del AG* has association with early age onset of breast cancer and exon specific selection of cohort for direct sequencing of different mutational analysis is a useful approach to be applied on *BRCA* analysis. Moreover it is suggested that *BRCA* analysis should also be meant for determination of treatment strategies and breast cancer Pakistani patients diagnosed with age less than forty should be screened for *BRCA1-185 Del AG* as a part of routine diagnostic marker before treatment.
